# Influence of ACPA‐positive rheumatoid arthritis on visual field testing in patients with arterial hypertension: A comparative cross‐sectional study

**DOI:** 10.1111/opo.12838

**Published:** 2021-06-02

**Authors:** Jascha Wendelstein, Barbara Fuchs, Sarah Schlittgen, Robert Zielke, Jeanette Brünner, Matthias Bolz, Rielke Alten, Carl Erb

**Affiliations:** ^1^ Department of Ophthalmology and Optometry Kepler University Hospital Linz Austria; ^2^ Medical Faculty Johannes Kepler University Linz Linz Austria; ^3^ Department of Oncology Clinical Centre of Havelhöhe Berlin Germany; ^4^ Group Practice for General Medicine Jüterbog Jüterbog Germany; ^5^ Eye Clinic at Wittenbergplatz Berlin Germany; ^6^ Department of Rheumatology Schlosspark Klinik Berlin Germany

**Keywords:** arterial hypertension, perimetry, rheumatoid arthritis, visual field

## Abstract

**Purpose:**

To evaluate a possible influence of anti‐cyclic citrullinated peptide autoantibodies (ACPA) ‐ positive rheumatoid arthritis (RA) on visual field (VF) testing in patients with arterial hypertension (aHT).

**Methods:**

We conducted an observational cross‐sectional study comparing patients with ACPA‐positive RA and aHT, patients with aHT and healthy subjects. Further inclusion criteria were visual acuity (VA) of 0.8 or better and age between 40 and 60 years. VF testing was performed with standard automated achromatic perimetry (SAP), short wavelength automated perimetry (SWAP) (Octopus 300^®^) and flicker perimetry (Pulsar^®^). Results were analysed for a possible correlation with blood pressure or RA‐activity.

**Results:**

Twenty subjects with RA and aHT, 26 patients with aHT and 22 healthy participants were examined. Significant differences were found for mean sensitivity (MS) in SWAP comparing RA‐patients with healthy participants (ΔMS −3.06, *p* = 0.001) and with hypertensive patients (ΔMS −2.32, *p* = 0.007). In SAP we observed a significant difference between patients with RA and healthy subjects regarding loss variance (LV) (ΔLV = +9.77, *p* = 0.004). Flicker perimetry did not demonstrate significant differences between groups. A correlation of VF changes with blood pressure level or RA‐activity was not observed.

**Conclusion:**

Patients with ACPA‐positive RA and aHT showed significant impairment of VF performance in SWAP compared to patients with aHT alone and healthy subjects. SAP also revealed a significant difference of LV between RA‐patients and healthy subjects. aHT does not seem to induce functional changes in VF testing alone.

## Introduction

Systemic comorbidities are very common in elderly ophthalmology patients, although they might not be the actual reason for a consultation. Arterial hypertension (aHT) has become a worldwide public health challenge, particularly in patients over 50 years of age. According to recent epidemiological data, 55% of the German population is affected by this condition.^1^ Moreover, there appears to be a rising incidence of chronic systemic autoimmune disorders like rheumatoid arthritis (RA), which is likely due to environmental factors.^2^

Rheumatoid arthritis is primarily characterised by inflammation and swelling of multiple synovial joints. However, extra‐articular organ manifestations such as heart, lung, kidney, skin and eye involvement can occur either at the beginning or during the course of the disease. Ophthalmic involvement is very common in chronic inflammatory rheumatic diseases and can, in principle, affect every part of the eye.^3^ The prevalence of aHT in these patients is even higher and is associated with accelerated atherosclerosis.^4^, ^5^ This fact is related to the systemic inflammation, the common use of corticosteroids and to disease‐induced physical inactivity in these individuals. Inflammation is associated with elevated blood levels of C‐reactive protein, which itself leads to reduced production of vasodilatative nitric oxide next to a higher expression of AT‐I receptors resulting in hypertensive dysregulation.^6^, ^7^

Interestingly, we still know very little about how these two common comorbidities affect visual physiology. Indeed, morphological changes of the retina in association with aHT have been identified clinically in funduscopy and described in the classification of hypertensive retinopathy.^8^ Nevertheless, the impact of the related microcirculatory hemodynamic disturbances and RA‐associated inflammatory changes on retinal sensory cells and their function has not been investigated thoroughly. We assume that a form of functional impairment occurs in retinal sensory cells when blood and oxygen supply are limited in atherosclerotic microvessels. To prevent cells from experiencing this undersupply, capillary vessels undergo an autoregulatory mechanism (described as the Bayliss effect) in the form of smooth muscle reaction to change and adaptation of the diameter of the vessel.^9^ This ensures continuous blood flow in capillaries even when blood pressure varies. Outside a given range of blood pressure, this autoregulated mechanism is overstrained and vessels widen in a more passive manner.

Endothelial dysfunction caused by chronic hypertensive conditions resulting in atherosclerotic vascular changes could impede microhemodynamics in retinal vessels.^8^ Critical undersupply of nutrients and oxygen to retinal cells may lead to functional disturbances measurable with perimetry. This effect has been described previously in patients with coronary heart disease and RA patients showing significant differences in perimetric outcomes when compared with healthy subjects.^10^, ^11^ Furthermore, impairment of colour vision in patients with arterial hypertension may be due to the same mechanisms.^12^ In patients with both RA and aHT, we can expect severe vascular damage. Reduced ocular perfusion is the logical consequence, which will lead to disabilities in retinal sensory cells and impairment of the visual field (VF). VF testing may reveal functional deficiencies of these cells, and clinicians can draw conclusions as to how it is affecting the condition of the ocular and systemic vessels. In a previous study, Schröder *et al*. did not show changes in white‐on‐white perimetry function in patients with aHT alone.^13^ While the display of the parvocellular system through standard automated achromatic perimetry didn’t seem to be suspicious, the question remains whether the detection of early damage in the magnocellular system is possible with more sensitive methods such as flicker perimetry or short wavelength automated perimetry.

Therefore, the aim of this study was to investigate whether early retinal perfusion related damage to the VF can be detected through flicker perimetry or short wavelength automated perimetry, and if RA as an additional vascular risk factor enhances these VF defects in a linear or nonlinear manner. A secondary aim is to explore a possible correlation between perimetric examinations, RA disease activity and blood pressure. We assume that morphological retinal changes occur very late in the disease process, and so functional visual examinations can be used for the early diagnosis of vascular comorbidities.

## Methods

The study protocol was approved by the Clinical Ethics Committee of the medical faculty, Charite University, Berlin and adhered to the tenets of the Declaration of Helsinki. The recruitment of all subjects was conducted at the Hospital Schlosspark Klinik, Berlin in the Department of Internal Medicine and Rheumatology and via a bulletin to recruit the control group. A patient information sheet and a consent form were signed by all participants.

A total of 68 patients who fulfilled inclusion and exclusion criteria were included in this cross‐sectional observational study. Each participant was allocated to one of three groups: 20 patients with RA and aHT, 26 patients with aHT solely and 22 control subjects. The distribution of female and male subjects in our groups was unequal in favour of female subjects, particularly in the rheumatoid arthritis cohort. Testing was performed on one eye per patient, which was either chosen at random if visual acuity (VA) was equal in each eye, or the eye with better VA was selected. Demographics are displayed in *Table* *1*.

**Table 1:** Classification of patient groups and patients’ characteristics

	Number	Sex (female:male)	Mean age ± S.D.	MAP (mmHg)	Systolic pressure (mmHg)	Diastolic pressure (mmHg)
RA + aHT	20	18:2	55 ± 4.3	102 ± 9	136 ± 13	85 ± 8
aHT	26	18:8	52.8 ± 4.8	103 ± 13	134 ± 18	87 ± 12
Control group	22	13:9	52.4 ± 2.9	94 ± 8	124 ± 12	79 ± 7

aHT, arterial hypertension; MAP, mean arterial pressure; RA, rheumatoid arthritis; S.D., standard deviation.

All subjects were between 40 and 60 years of age, in order to achieve a homogeneous patient collective. With increasing age, loss of accommodation as well as cloudiness of the optical media such as age‐related cataract leading to reduced VA and contrast are anticipated; these confounding variables were excluded by the age restriction and ocular examination. The fundamental precondition for the aHT group was aHT requiring pharmaceutical treatment. Inclusion in the RA group required a confirmed diagnosis based on the classification criteria of the American College of Rheumatology (ACR), the presence of anti‐cyclic citrullinated peptide autoantibodies (ACPAs) and coexistent aHT with medication‐based treatment.

In order to avoid bias effects, exclusion criteria were other comorbidities and risk factors leading to vascular changes such as peripheral arterial occlusive disease, current smokers or cessation of smoking within one year, hyperlipidemia, coronary heart disease, stroke history, diabetes, chronic obstructive pulmonary disease and substance abuse (dependence on alcohol or drugs). We used the Cut Down‐Annoyed‐Guilty‐Eye Opener (CAGE) questionnaire adapted to include drug use (CAGE‐AID) as an anamnestic screening tool for detecting problematic consumption at a cut‐off grade of 2 or higher.^14^ Ophthalmological exclusion criteria included severe visual impairment or following anomalies: refractive errors <−3.00 and >+3.00 dioptres, corrected VA > logMAR 0.1, history of glaucoma, intraocular pressure > 21 mmHg, cup‐disc‐ratio > 0.6 in a normal sized optic nerve, recurring episodes of uveitis, eye surgery and scotoma of other cause. Participants in the aHT group were excluded if any chronic systemic autoimmune disease was prevalent. Exclusion criteria for the control group were aHT or any long‐term medication.

Distance VA was measured through the manifest refractive correction determined in a standardised ophthalmological examination to ensure subjects met the exclusion criteria. This examination included measurement of intraocular pressure (Goldmann applanation tonometry) and a clinical slit lamp examination with funduscopy to check for the presence of any abnormalities specified as exclusion criteria.

Rheumatologic examination and disease activity evaluation were conducted on patients in the RA‐group. As recommended by the ACR for clinical practice, rheumatic disease activity was calculated using the following score systems: DAS‐28 (Disease Activity Score 28) and CDAI (Clinical Disease Activity Index). In addition, blood samples were taken to measure ACPAs and other inflammatory parameters and blood pressure was measured using the Riva‐Rocci method).

The aim of this study was to compare and correlate VF indices such as mean sensitivity (MS) and loss variance (LV) with three perimetric techniques in the three cohorts namely aHT, rheumatoid arthritis in combination with aHT and a control group.

Patients underwent three different visual field techniques within the central 30 degrees. Testing was performed in random order to prevent a sequence effect. The ‘randbetween’ function in Excel 2007^®^ (Microsoft, www.microsoft.com) was used as the randomisation method to allocate each patient to one of six groups, which included each possible sequence for the three measurements. The tests were performed by the same trained personnel. To mitigate against any bias rising from a learning effect, all participants performed three other visual field tests before the actual experimental testing. Sufficient time was allowed between the tests to reduce the effects of fatigue and stress. Tests were termed reliable with <20% fixation losses, false positives and false negatives.

Standard automated achromatic perimetry was performed using the SAP, Octopus 300 (Haag‐Streit, www.haag‐streit.com). Using the Octopus 300 dG1 program, the threshold for the local differential luminance sensitivity (DLS) was assessed, which took 4 to 6 min to complete. Mean sensitivity (MS) was described as the average of the threshold limit values in the VF. Values were recorded in decibels and indicated diffuse distributed retinal deficiencies in the VF. Loss variance (LV) was described as the variance of deviations from the age‐related standard and was a measure for visual defect irregularity. Individual defects that represented strong outliers would lead to pathological LV values.

Flicker perimetry was performed using the Pulsar (Haag‐Streit, www.haag‐streit.com). Sixty six moving flicker stimuli having different frequencies were presented to measure the temporal visual function, spatial resolution and contrast sensitivity. Flicker perimetry was performed with a background luminance of about 31.8 cd m^−2^ and a 500 ms stimulus duration. The amplitude of flicker decreased towards the periphery. Again, outcome values were MS and LV.

Short wavelength automated perimetry (SWAP) or blue‐on‐yellow perimetry was carried out using the Octopus 300 (Haag‐Streit, www.haag‐streit.com). Using the Octopus 300 cG1 programme, blue stimuli on a yellow background selectively stimulated the blue cones. SWAP testing was conducted with a background luminance of 100 cd/m^2^ and a 200 ms stimulus duration. The setup and procedure for this perimetric technique was similar to the SAP measurement. Again, outcome parameters were MS and LV. LV values were obtained by squaring the machine‐generated square root of loss variance (sLV), which was defined as the standard deviation of the measured deviations from the age‐related standard for all three perimetric examinations.

### Statistical analysis

Statistical analysis was carried out using Excel 2007^®^ (Microsoft, www.microsoft.com), R^®^ (Version 2.12.1, The R Project for Statistical Computing, www.r‐project.org/) and SPSS (Version 22.0, IBM, www.ibm.com). *p*‐values less than 0.05 were considered as statistically significant.

The Shapiro‐Wilk test was used to determine whether a data set was normally distributed. Descriptive statistics were presented as mean and standard deviation (S.D.) for normally distributed variables, and median and mean absolute deviation about the median (MAD) for non‐normally distributed variables. Given a normal distribution with equal variances, perimetric variables were compared using one‐way anova and post hoc t‐test. When comparing the three groups, normally distributed variables with equal variances were compared using one‐way anova and post hoc unpaired t‐test. Variables with unequal variances were compared using the non‐parametric Kruskal‐Wallis test. We applied the Mann‐Whitney‐U test for independent samples and the Wilcoxon test for dependent samples.

Nominal variables were compared using the χ^2^ test. Pearson correlation for normally distributed samples and Spearman correlation for unequally distributed samples were used to assess the relationship between disease activity or blood pressure and perimetric parameters. To counteract the family‐wise error‐rate of multiple comparisons, the Bonferroni‐Holm correction method was applied.

## Results

### Clinical results

Investigation outcomes were assessed and analysed for a total of 68 patients fulfilling the inclusion and exclusion criteria, divided into three observational groups as summarised in *Table* *1*. No significant differences in age or ophthalmological characteristics were observed between the three groups.

Seven out of 26 aHT patients and five out of 20 RA patients, demonstrated degree I hypertensive fundus changes based on the classification of Keith‐Wagener.^8^ Marginal constrictions and coils of the retinal arteries were noted. After correction with the Bonferroni‐Holm method, no significant differences in mean arterial pressure (MAP), systolic and diastolic blood pressure were seen between the groups.

The mean (S.D.) duration of aHT was comparable in the AhT [7.6(7.6) years] and RA [9.8(8.1) years] cohorts. Therapeutic control of arterial hypertension was also similar as shown in *Table* *2*. The mean (S.D.) duration of rheumatoid disease in the RA group was 11.2 (9) years. The therapeutic regimen was sub‐categorised, as shown in *Table* *2*.

**Table 2:** Medical therapeutical regimen of arterial hypertension (aHT) and rheumatoid arthritis (RA)

	aHT	RA with aHT
Therapy of aHT (medication)		
Monotherapy	11	11
Twofold combination	10	7
Threefold combination	5	2
Therapy of RA (medication)		
Glucocorticoids		1
Synthetic DMARD (no MTX)		3
MTX		10
MTX with glucocorticoids		2
Biological DMARD		4

DMARD, disease‐modifying anti‐rheumatic drug; MTX, methotrexate.

Mean (S.D.) relative elevation level of ACPA in the RA cohort was 31.58 (42.62). Sixty percent of patients were sero‐positive for rheumatoid factor, 20% were sero‐negative and 20% had unknown status. With regard to disease activity in the RA patients, the commonly used activity scales DAS‐28 and CDAI revealed that the majority of the patient collective had a moderate to high disease activity [DAS‐28: 55% moderate activity, mean 4.9 (1.8); CDAI: 70% high activity, mean 29.3 (18.6)].

### Perimetric results

In all three perimetric strategies, no significant differences between groups were observed for the secondary indices (i.e., reliability, false/negative errors, duration of examination). *Table* *3* shows the main perimetric indices MS and LV for the three groups.

**Table 3:** Results of visual field indices per group

	aHT	RA with aHT	Control group	*p* value KW/VA	*p* value aHT vs C	*p* value aHT vs RA	*p* value RA vs C
	mean ± S.D., median (MAD median)						
SAP							
MS (dB)	27.7 ± 1.4 27.6 (1.0)	26.8 ± 1.9 26.9 (1.6)	28.0 ± 0.9 28.1 (0.7)	0.10			
LV (dB)	6.19 ± 5.3 3.8 (4.1)	13.2 ± 18.0 6.4 (11.4)	3.4 ± 1.5 3.3 (1.2)	**0.004**	0.13	0.13	**0.004**
SWAP							
MS (dB)	21.0 ± 2.3 21.5 (1.7)	18.7 ± 3.3 18.8 (2.6)	21.8 ± 2.0 21.4 (1.6)	**<0.001**	0.33	**0.007**	**0.001**
LV (dB)	16.7 ± 9.7 13.1 (7.5)	20.5 ± 11.1 18.0 (8.3)	13.7 ± 7.4 10.7 (5.5)	**0.05**	0.27	0.25	**0.05**
FP							
MS (dB)	18.9 ± 2.6 19.7 (1.7)	19.0 ± 2.4 19.4 (2.0)	19.3 ± 1.3 18.9 (1.0)	0.94			
LV (dB)	2.4 ± 1.2 2.3 (0.8)	2.3 ± 1.2 2.4 (0.9)	2.2 ± 0.9 2.3 (0.7)	0.96			

aHT, arterial hypertension; C, control group; FP, flicker perimetry; KW, Kruskal‐Wallis test; LV, loss of variance; MAD median, mean absolute deviation about median; MS, mean sensitivity; RA, rheumatoid arthritis; SAP, standard automated achromatic perimetry; S.D., standard deviation; SWAP, short wavelength automated perimetry; VA, variance analysis.

*p*‐values < 0.05 shown in bold.

**Figure 1:**
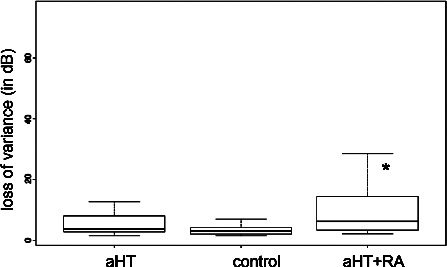
Comparison of loss variance (LV) values in standard automated achromatic perimetry between groups: RA patients with aHT (*) demonstrated a significant augmentation in LV compared to aHT alone and to control.

Comparison of the non‐normally distributed MS and LV values of SAP across groups with the Kruskal‐Wallis test revealed no significant difference in MS. However, a significant increase in LV was observed with in the RA group compared with the controls (ΔLV = +9.77; Wilcoxon test *p* = 0.004) (*Table* *3*, *Figure* *1*). Further, the increase in LV seen in the RA group was weakly correlated with DAS‐28 (*r* = 0.32, *p* = 0.15), but not with systolic (*r* = 0.10, *p* = 0.68) or diastolic blood pressure (*r* = 0.16, *p* = 0.50).

**Figure 2:**
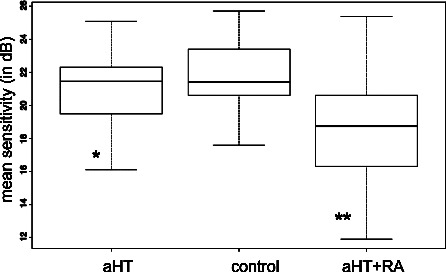
Comparison of mean sensitivity (MS) values in short wavelength perimetry between groups: RA patients with aHT demonstrated a significant augmentation in MS (**) compared to aHT (*) alone and to control.

**Figure 3:**
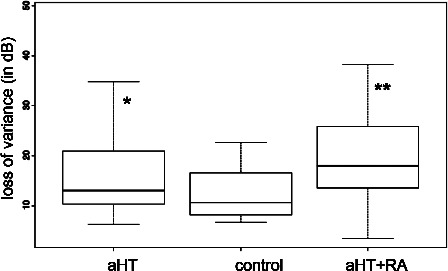
Comparison of loss variance (LV) values in short wavelength perimetry between groups: RA patients with aHT demonstrated a significant augmentation in LV (**) compared to aHT (*) alone and to control.

With SWAP, we found a significant difference in normally distributed MS values with variance analysis. The post hoc paired t‐test revealed significant differences in the RA group compared to the control (ΔMS −3.06, *p* = 0.001) and aHT groups (ΔMS −2.32, *p* = 0.007). The Kruskal‐Wallis test was used for non‐normally distributed SWAP LV values which showed a significant effect. Hence, the Wilcoxon test was performed, which revealed a significant difference in the RA group compared to the controls (ΔLV = +6.81 dB, *p* = 0.05) (*Table* *3*, *Figures* *2* and *3*). In particular, a globally reduced mean retinal sensitivity was observed in the RA group as well as an increase in loss variance values. These differences were not found when comparing the aHT and control groups. No significant correlation was detected for disease activity indices and blood pressure. In flicker perimetry, no significant between‐group differences were detected for the non‐normally distributed MS and LV values using the Kruskal‐Wallis test (*Table* *3*).

### Statistical results

Effect sizes of standardised mean differences are widely used for power‐analysis to calculate the statistical power after the study has been conducted. Here, the significance level α was 5%, sample size was 68 and effect size was 0.586, so post hoc power of MS values after SWAP was 99%. This post hoc power analysis was useful to estimate variation within the population and to aid future experimental design.

## Discussion

Visual field examinations are an integral part of clinical practice, predominantly for diagnosing pathological conditions of the retina, optic nerve and optic tract/cortex. Since systemic disorders can affect the function of these anatomical structures, our aim was to characterise the influence of aHT both as a distinct disease entity as well as a comorbidity of inflammatory RA using different perimetric methods.

Diagnostic certainty in our RA patient collective was increased by including only ACPA‐positive subjects, which is a surrogate marker with a high specificity of 95%.^15^ The presence of ACPAs is associated with a higher cardiovascular risk and accelerated atherogenesis.^16^, ^17^

Further, having a higher proportion of female subjects in the RA group is consistent with the epidemiology of many rheumatic diseases, including RA, which are more frequent in females than males. The female/male ratio in the RA group studied here was 18/2, which is a greater gender difference compared with the proportions reported previously for this disease. In general, the incidence is 3‐4 times higher in females, but it depends on many factors, including a higher incidence in females approaching menopause and a decreasing female/male ratio after the age of 60. All of the subjects tested here were between 40 and 60 years of age, which could explain the greater gender difference in the RA group, but the sample size is too small to allow further interpretation.^18^

In the RA group, SAP results demonstrated a significant rise in LV, which was weakly correlated with disease activity (DAS‐28). Therefore, this cohort showed more localised deep defects in SAP than the control and aHT groups. In order to interpret this observation, it is necessary to remember that SAP measures a broad cell community and is functionally dependent on the parvocellular and magnocellular ganglion cell systems. This determines the relatively low sensitivity of this method, identifying defects only after a loss of 25‐50% of the ganglion cells.^19^ Large ganglion cells receptive fields can compensate for the defective ganglion cells for a long time. Thus, the general light sensitivity threshold is altered at a late stage by these compensatory mechanisms, which makes early dysfunction undetectable with this particular diagnostic technique. However, these compensation mechanisms were not sufficient in the RA patients, so that the measured defects became even more evident when using more specific VF examination techniques such as SWAP. In addition, other advanced imaging technologies such as fluorescence angiography or optical coherence tomography would be an interesting comparison to investigate in the context of this topic. Further studies are necessary to identify the most sensitive method for detecting measurable disease in patients with RA.

No correlation was seen between the higher LV with SAP in RA patients with blood pressure levels. One explanation might be due to the efficient blood flow autoregulation in the retinal and chorioidal vessels^20^ of our aHT cohort, patients did not exceed autoregulatory limits with their MAP levels. Additionally, none of the patients showed moderate or severe forms of hypertensive retinopathy due to their therapy regimes. We assume that all antihypertensive medications have a protective vascular effect, thereby preventing VF damage.

While SAP was only capable of revealing slight differences in perimetric indices, SWAP was able to demonstrate significant differences in the main MS and LV indices in RA patients. This was not surprising because this technique assessed the functionality of blue cones and the downstream localised koniocellular ganglion cells. These are a small neuronal cell population of the human retina (10% of all retinal ganglion cells) and a highly specialised cell system that selectively perceives blue‐yellow stimuli.^21^, ^22^, ^23^ Due to the specific stimulus, the main advantage of this method is the early detection of functional disturbances (3–5 years in advance of SAP).^24^, ^25^ The main disadvantages of SWAP compared with SAP are that localised scotoma are detected less precisely, examination time is longer and results are more age‐dependent.^26^

Finally, the results from flicker perimetry did not demonstrate significant changes in perimetric indices between the groups. Flicker perimetry also belongs to the group of new, non‐conventional perimetric tests providing earlier detection of VF damage by selectively stimulating certain visual pathways to overcome potential redundancy.^27^, ^28^ Flicker perimetry, like SAP, is dependent on the function of parvo‐and magnocellular ganglion cells, but is capable of testing temporal and spatial contrast sensitivity functions simultaneously.^28^ The flicker test is a rapid perimetric procedure that is easy and comfortable for patients.^29^ Multiple studies have reported higher sensitivity when detecting early primary open‐angle glaucoma using flicker perimetry.^24^, ^30^, ^31^ In the present study, this perimetric strategy was not sufficiently sensitive to detect VF disturbances revealed by SWAP in RA patients. This may be attributable to a greater overlap of the tested cellular pathways and therefore elevated redundancy potential in flicker perimetry. Moreover, there are other differences between flicker perimetry and SWAP including the test stimulus duration and the measured values, which can influence these perimetric results. While the ideal perimetric testing should be accurate, efficient and repeatable, it remains a subjective and situational examination. This has to be considered when reflecting the underlying neural damage.^32^

Side effects of the numerous combination therapies in our aHT and RA patients on VF procedures were difficult to evaluate. We assume that conventional as well as biological disease‐modifying anti‐rheumatic drugs (DMARDs) and antihypertensive treatments exert a positive regulatory influence on microvessels. In contrast, non‐steroidal anti‐inflammatory drugs and glucocorticoids could worsen systemic blood pressure regulation and contribute to VF defects. It was not possible to eliminate the possible impact of medication on our results.

Currently, it is not possible to differentiate between VF disturbances caused by a primary ophthalmological disease and the overlapping effects of coexisting systemic diseases. Our findings demonstrated that ACPA‐positive RA patients with aHT showed VF anomalies with highly sensitive perimetric procedures such as SWAP. However, aHT alone did not affect SAP, SWAP or flicker perimetry. Further, no clinically relevant correlation between functional VF impairment and blood pressure level or RA disease activity was detected. Nevertheless, the extent to which functional impairment in sensitive perimetric procedures such as SWAP may be a diagnostic means of monitoring interdisciplinary disease remains to be elucidated. Furthermore, it remains evident that eye care practitioners must consider systemic disorders when interpreting perimetric results, although aHT does not appear to be a confounder in the perimetric methods tested here.

## Conflict of interest

The authors report no conflicts of interest and have no proprietary interest in any of the materials mentioned in this article.

## Author contributions

**Jascha Wendelstein:** Writing‐original draft (equal); Writing‐review & editing (equal). **Barbara Fuchs:** Writing‐original draft (equal); Writing‐review & editing (equal). **Sarah Schlittgen:** Conceptualization (equal); Data curation (equal); Formal analysis (equal); Methodology (equal); Software (equal); Visualization (equal). **Robert Zielke:** Data curation (equal); Investigation (equal); Resources (equal). **Jeanette Brünner:** Investigation (equal); Resources (equal); Supervision (equal); Validation (equal); Writing‐original draft (equal). **Matthias Bolz:** Supervision (equal). **Rieke Alten:** Data curation (equal); Investigation (equal); Methodology (equal); Resources (equal). **Carl Erb:** Conceptualization (equal); Project administration (equal); Resources (equal); Supervision (equal); Validation (equal).
